# *In silico* analysis of high affinity potassium transporter *(HKT)* isoforms in different plants

**DOI:** 10.1186/2046-9063-10-9

**Published:** 2014-09-15

**Authors:** Mahbobeh Zamani Babgohari, Esmaeil Ebrahimie, Ali Niazi

**Affiliations:** 1 Biotechnology Institute, Shiraz University, Shiraz, Iran; 2 Department of Crop Production & Plant Breeding, College of Agriculture, Shiraz University, Shiraz, Iran; 3 School of Molecular & Biomedical Science, The University of Adelaide, Adelaide, Australia

**Keywords:** *HKT*, Gene network, Promoter, Regulatory elements, *In silico* synteny, Pathway discovery

## Abstract

**Background:**

High affinity potassium transporters (HKTs) are located in the plasma membrane of the vessels and have significant influence on salt tolerance in some plants. They exclude Na^+^ from the parenchyma cells to reduce Na^+^ concentration. Despite many studies, the underlying regulatory mechanisms and the exact functions of *HKT*s within different genomic backgrounds are relatively unknown. In this study, various bioinformatics techniques, including promoter analysis, identification of *HKT*-surrounding genes, and construction of gene networks, were applied to investigate the *HKT* regulatory mechanism.

**Results:**

Promoter analysis showed that rice *HKTs* carry ABA response elements. Additionally, jasmonic acid response elements were detected on promoter region of *TmHKT1;5. In silico* synteny highlighted several unknown and new loci near rice, *Arabidopsis thaliana* and *Physcomitrella patent HKT*s, which may play a significant role in salt stress tolerance in concert with *HKTs*. Gene network prediction unravelled that crosstalk between jasmonate and ethylene reduces *AtHKT1;1* expression. Furthermore, antiporter and transferase proteins were found in *AtHKT1;1* gene network. Interestingly, regulatory elements on the promoter region of *HKT* in wild genotype (*TmHKT1;5*) were more frequent and variable than the ones in cultivated wheat (*TaHKT1;5*) which provides the possibility of rapid response and better understanding of environmental conditions for wild genotype.

**Conclusion:**

Detecting ABA and jasmonic acid response elements on promoter regions of *HKTs* provide valuable clues on underlying regulatory mechanisms of *HKTs. In silico* synteny and pathway discovery indicated several candidates which act in concert with *HKTs* in stress condition. We highlighted different arrangement of regulatory elements on promoter region of wild wheat (*TmHKT1;5*) compared to bread wheat (*TaHKT1;5*) in this study.

## Introduction

Under salinity stress, the uptake of Na^+^ into cells occurs through multiple Na^+^-permeable cation channels/transporters, such as outward and inward-rectifying K^+^-selective channels, in particular non-selective cation channels in the plasma membrane [1]. Loading of xylem vessels with Na^+^ results in its upward transportation via the transpiration system [2].This transport triggers ion toxicity when the cytoplasmic concentration of Na^+^ reaches to threshold level [2].

Little is known about Na^+^ excluding proteins in plants. *HKTs* are a large superfamily of transporters. They share sequential and functional similarities with the TrkH/KtrB group of cation transporters in bacteria and fungi [3,4]. It has been proposed that these transporters play crucial roles in salinity tolerant via removal of Na^+^ from the xylem during salinity stress [1,2].

*In silico* promoter analysis can produce valuable information about the function and signalling of a gene. The superiority of an *HKT* homologue to other homologues can actually be related to the superior promoter structure, rather than the gene structure. Regarding the unknown role of *HKT* promoters, *in silico* promoter analysis can provide valuable information. The regulatory elements in promoters, such as transcription factor binding sites, are organized into distinct modules that control expression in many genes. Thus, the identification of regulatory elements is vital for the recognition of gene expression patterns [5].

The conserved orientation of *HKTs* and surrounding genes on a chromosome has not been addressed in previous studies. Identification of comparative genetic maps through *in silico* synteny can provide the opportunity to acquire information about the evolution and function of a gene cluster via cytogenetic events. It should be noted that the specific orientation of genes in a particular region of a chromosome is commonly associated with particular functions of those genes [6].

In addition to promoter and *in silico* synteny, network discovery based on available transcriptomics data as well as text mining can be used to understand the function and regulatory mechanisms of *HKTs*. Construction of gene networks is a powerful tool in detection of genes involved in specific processes, such as biotic and abiotic stress [7]. Recognizing relationships between co-expressed genes and illustrating the involved pathways provide valuable clues on the effect and the role of gene of interest. However, the physiological functions, gene networks, and signalling pathways related to *HKT* transporters have not yet been completely clarified [8].

In the present study, bioinformatics analysis was employed to illustrate the functional pathways related to *HKT* transporters in plants and to discover the *HKT* homologues. The promoter regions of *HKT* isforms were analyzed. Moreover, *in silico* synteny was studied as the exact determination of orthology is significant in comparative genomics and biological processes. *HKT-*gene network was built for the first time using available microarray data in order to predict the interacting genes and their possible functions in the stress condition.

## Material and methods

### Promoter analysis

The sequences of all available *HKTs* in wheat, wild wheat relative (*Triticum monococcum)*, rice and *Physcomitrella patens* were downloaded from the NCBI (ncbi.nlm.nih.gov) database. The rice sequences were: *OsHKT2;1* (AB061311), *OsHKT2;2* (AB061313), *OsHKT2;3* (AJ491820), *OsHKT1;1* (AJ491816), *OsHKT1;3* (AJ491818), *OsHKT1;4* (AK120889), *OsHKT1;5* (EF373553), and *OsHKT2;4* (AJ491855). One kb upstream (from the start codon) of the genes were extracted as promoter sequences using Phytozome database ( http://www.phytozome.net/) and Osiris database ( http://www.bioinformatics2.wsu.edu/cgi-bin/Osiris/cgi/home.pl). As there was no available database for promoter identification in wheat or wild wheat, a thesis published by Byrt in 2008 was used for *TaHKT1;5* and *TmHKT1;5*[9]. The putative promoter sequences of *HKT* genes in rice, bread wheat and *T. monococcum* were compared with known cis-regulatory elements in the collection of the PLANT CARE database ( http://bioinformatics.psb.ugent.be/webtools/plantcare/html/). The cis-regulatory elements were listed and counted for each promoter. Promoter sequences of rice *HKTs* were also analyzed through the Osiris database where we used rice *HKTs* accession numbers to find the transcription factor binding sites across the promoter regions. Using this database, significant regulatory elements were selected at the 0.05 probability level (based on Fisher’s exact test) to discriminate the transcription factors which have high binding possibility to promoters.

### Finding neighbouring genes (synteny analysis)

Most of the genomic data, stored and publicly available in EMBL and NCBI databases, are without extensive synteny visualization tools [6]. *HKT* orthologs, initially compiled from BLAST searches of sequences of *Arabidopsis thaliana*, rice and *physcomitrella patent*, were extracted from the phytozome database (phytozome http://www.phytozome.net). A synteny-based approach was used to identify genes adjacent to *HKTs*. In addition, up to 15 genes up and downstream of rice *HKTs* were detected using the Gramene database ( http://www.gramene.org/genome_browser/index.html).

### Gene network discovery for *AtHKT1;1*

In this study, 2 sources of microarray data were retrieved for analysis:

### Single selected microarray experiment from “Plant Expression Database”

Micoarray experiment was selected from “Plant Expression Database” ( http://www.plexdb.org/). At first, probeset ID of *AtHKT1;1* (255812_at) was retrieved from affymetrix database ( http://www.affymetrix.com/estore/). Then, different deposited microarray experiments in “Plant Expression Database” were mined using *AtHKT1;1* probeset ID. Finally, microarray experiment (Microarray ATH1-121501) considering cross-talk between jasmonate and ethylene signalling in *Arabidopsis* seedlings was selected. In this experiment, 3 Arabidopsis strains (Col-0, coi1-2, and ein3eil1) were treated by Mock and MeJA. The reason for choosing this experiment was that it included two hormones, which could unravel *HKT* expression pattern, its coexpressed genes, and its genetic interaction network.

Then, the data of this experiment was analyzed using pathway studio 9 and ResNet5.0 database. Pathway Studio is a commercial product for pathway analysis, containing a comprehensive database of protein–protein relationships extracted from literature using MedScan an entirely automated biomedical information extraction engine [10].

### Multiple microarray experiments extracted from ATTED-II database for co-expression network analysis

The co-expression of genes involved in the *AtHKT1;1* process was also explored in ATTED-II database ( http://atted.jp/) [11]. Source of GeneChip data in ATTED-II database is TAIR ( http://arabidopsis.org/index.jsp). For construction of co-expressed network of *AtHKT1;1*, 58 microarray experiments and 1388 array slides were used. This database collects gene expression data in Arabidopsis from a wide range of microarray experiments. ATTED-II database employs Mutual Ranking (MR score) for co-expresstion analysis instead of Pearson correlation test. Co-expressed of *HKT1;1* in abiotic, biotic, hormone and light experiments were extracted [12,13].

### Comparative statistics of regulatory elements on promoter regions of *TaHKT1;5* (bread wheat) and *TmHKT1;5* (wild wheat)

To have a better understanding of differential arrangement of regulatory elements on the promoter region of *HKT* in bread wheat verses its wild relatives, the predicted regulatory elements (Table 1) were compared between *TaHKT1;5* and *TmHKT1;5* in both aspects of central and variation tendencies.

**Table 1:** **Elements present in the promoter region of *TaHKT1;5 *** **(bread wheat) and *TmHKT1;5 *** **(wild wheat) according to the PLANT CARE database**

**cis element**	***TmHKT1;5***	***TaHKT1;5***	**Function**
CAAT-box	12	7	Common cis-acting element in promoter and enhancer regions
Circadian	2	2	cis-acting regulatory element involved in circadian control
CATT-motif	2	2	Part of a light-responsive element
AE-box	0	1	Part of a module for light response
G-box	2	1	cis-acting regulatory element involved in light responsiveness
MRE	0	1	myb binding site involved in light responsiveness
LTR	2	2	cis-acting element involved in low-temperature responsiveness
TC-rich repeats	1	1	cis-acting element involved in defence and stress responsiveness
ARE	1	0	cis-acting regulatory element essential for the anaerobic induction
Box 4	1	1	Part of a conserved DNA module involved in light responsiveness
Box I	1	1	Light-responsive element
ERE	1	1	Ethylene-responsive element
HSE	1	2	cis-acting element involved in heat stress responsiveness
Skn-1_motif	1	1	cis-acting regulatory element required for endosperm expression
TATA-box	22	18	Core promoter element approximately −30 of the transcription start
ACE	1	1	cis-acting element involved in light responsiveness
I-box	2	0	Part of a light-responsive element
MNF1	1	1	Light-responsive element
O2-site	1	0	cis-acting regulatory element involved in zein metabolism regulation
ATCT-motif	1	1	Part of a conserved DNA module involved in light responsiveness
CCAAT-box	1	1	mybHv1 binding site
CGTCA-motif	2	1	cis-acting regulatory element involved in the MeJA-responsiveness
Sp1	2	3	Light-responsive element
TGA-element	1	1	Auxin-responsive element
TGACG-motif	2	1	cis-acting regulatory element involved in the MeJA-responsiveness
GARE-motif	1	1	Gibberellin-responsive element
CCGTCC-box	0	1	cis-acting regulatory element related to meristem specific activation
A-box	0	1	cis-acting regulatory element

## Result and discussion

### Comparative Study of Promoter Regions between *HKT1;5-A* and *HKT1;5-D*

Many studies have shown that *HKT* expression is different among various cell types of plants [2,14]. Moreover, different isoforms of *HKT* function differently. For instance, it has been reported that *TmHKT1;5-A* (*HKT* isoform in *Triticum monococcum*) decreased Na^+^ concentrations in leaf blades and sheaths to a greater extent than *TmHKT1;4-A*[2]. In our previous study [15], we measured expression pattern of *HKT1;5* in wheat and its wild relatives (A and D genomes) under salt stress using quantitative-real time PCR technique. The results confirmed that *HKT1;5* expression is tissue and genotype dependent [15]. In the mentioned study, earlier expression of *HKT1;5* in response to salt stress was observed in leaves of bread wheat rather than roots. Additionally, under high NaCl level (200 mM) treatment, the allele of D genome (*HKT1;5-D*) showed higher expression than the allele of A genome (*HKT1;5-A*). We found that promoter region of *HKT1;5-D* has jasmonic acid response element [15] which this element is not present at *HKT1;5-A*. In other words, the observed differential expression pattern of *HKTs* can be explained by differential architecture of promoter regions in the view of existence/absence of regulatory elements. This is an encouraging and mostly unknown topic for future studies.

The results of the PLANT CARE analysis of promoter regions are shown in Table 1. Interestingly, TATA box and CAAT elements were more frequent in the *TmHKT1;5* promoter than *TaHKT1;5.* The presence and the number of TATA boxes can increase the quantity of gene expression. However, the exact activation of the putative TATA boxes in *HKTs* has not been investigated in laboratory level.

Some studies suggested that TATA box has a variable position in −100 and −40 nucleotides from the start codon [16-20]. In contrast, other studies argued that the effective region of TATA box is 32 ± 7 and can alter up to 50 nucleotides upstream of the transcription initiation site [16-19]. It seems that position of putative TATA box is variable between different gene promoters.

Interestingly, Sharma and co-workers in 2011 illustrated that the presence of two TATA boxes at positions −59 and −359 and two CCAAT elements sitting at positions of −355 and −590 are involved in strong expression of *AlX* in *Aspergillus Niger*[21].

Accordingly, it is possible that each of the predicted TATA boxes in the present study to have positive impact on *HKT* expression regardless of their positions on the promoter region. Consequently, we supposed that the increased number of TATA boxes in promoter of *TmHKT1;5* (wild wheat) compared to *TaHKT1;5* (bread wheat) can result in higher expression of *TmHKT1;5*.

PLANTCARE analysis detected high frequency of TATA boxes around position −50 (−42/ -44/ -50/-52) in *TmHKT1;5* while in *TaHKT1;5* promoter, the peak of TATA boxes happened around position −30 (−19/ -20/ -22/ -35). In our 2012study, we suggested that in high salinity condition, D genome of *TaHKT1;5* is more effective than A genome of *TbHKT1;5* and *TmHKT1;5*[12]. With respect to the position of TATA boxes in *TaHKT1;5* promoter (around position −30), it can be concluded that position of −30 is more important than position of −50 in initiation of transcription under high salinity (200 mM condition), conferring higher level of expression in *TaHKT1;5* compared to *TbHKT1;5*. In other words, it seems that at least under high salinity stress, the number of TATA boxes in a specific position of promoter (−30) is more effective than the whole numbers of TATA boxes which are spread across the entire promoter region. It should be noted that TATA boxes in other regions of promoter such as position of −50 in *TmHKT1;5* can function in different situations such as other abiotic/biotic stresses. The link between position of TATA box and tissue specific expression is also probable as *TmHKT1;5* has significantly higher expression in roots rather than the leaves [22].

Another interesting regulatory element in comparison of *TmHKT1;5* with *TaHKT1* is CAAT-box (Table 1). CAAT-box sequences have proved positive impact on frequency of transcriptional initiation [23]. Interestingly, *in silico* based analysis of promoter regions in this study revealed the existence of two CAAT-boxes in *TmHKT1;5* promoter at the range of −50 to −100 position (−53 and −76, respectively). In contrast, CAAT-boxes in promoter of *TaHKT1;5* are located out of −100 position from the start codon. Arrangement of CAAT-boxes close to transcription start codon in *TmHKT1;5* can help wild wheat in better response to environmental signals trough rapid activation of *HKT1*.

Since many defensive genes respond to jasmonate, jasmonates are universal signals of defense-related gene expression [24]. In the present study, the jasmonate response element (MeJA) was two times more common in *TmHKT1;5* than *TaHKT1*;5 (Table 1). Jasmonate is involved in plant adaptations to biotic and abiotic stresses, and is accumulated transiently in response to osmotic/salt stress [25].

It has been stated that expression of *TbHKT1;5* is higher than *TaHKT1*;5 under low/medium salt stress (50 and 100 mM NaCl) [15]. So, this salinity tolerance in the *TbHKT1;5* can be related to some elements in the promoter region such as jasmonate response element (MeJA) that was more frequent in this plant. Interestingly, it has been shown that application of MeJA improves the tolerance under moderate saline stress (40 mM NaCl) in broccoli, but not in high salinity conditions (200 mM NaCl) [26], which is in parallel to our assumption about the role of MeJA in *TmHKT1;5*/ *TbHKT1;5* promoter. It is possible that the MeJA and TATA boxes are not active in higher salt conditions, so the expression of *HKT* decreases in *T.boeoticum* in high salinity condition. However, the important point is that jasmonate response element can cause salinity tolerance, a positive trait in salt environments at least in low/moderate salinity conditions.

The promoter analysis also showed that HSE, a cis-acting element involved in heat stress responsiveness, is more frequent in *TaHKT1;5-D* than *TmHKT1;5*. Thus, it is likely that these *HKT*s are expressed in response to high temperature too.

### Promoter analysis of all *HKT* isoforms in rice

The promoter analysis was performed in all available rice *HKT* transporter isoforms, separated into two subfamilies. There are two subfamilies of *HKTs* based on glycine or serine substitution of a residue predicted in the first pore loop of the protein [27,4]. The results are divided into two tables based on subfamilies I and II (Table 2 and Table 3). In subfamily I, HKT transporters have a serine residue that determines Na^+^ transport, but subfamily II contains a glycine residue, responsible for K^+^ permeability primarily [9,14,28].

**Table 2:** **Elements present in the promoter regions of subfamily I of *HKTs *** **in rice according to the PLANT CARE database**

**Function**	**Motif**	***HKT1;1***	***HKT1;3***	***HKT1;4***	***HKT7*** **1;** ***4***	***HKT81;5***
		**AJ491816**	**AJ491818**	**AK10985**	**AK120889**	**AK108663**
ABA	ABRE	1	-	2	2	-
	IIB	-	-	1	1	-
	CE3	-	-	1	1	-
Salicylic acid	TCA	2	1	-	-	-
Ethylene	ERE	-	-	-	-	-
Auxin	AuxRR-core	2	-	-	-	-
	TGA	1	-	-	-	-
Gibberellin	P-Box	-	-	-	-	-
	GARE	-	-	-	-	1
MYB	MBS	2	2	1	1	1
Defence&stress	TC-reach	-	1	2	2	1
Heat stress	HSE	3	3	-	-	-
Low temperature	LTR	2	-	-	-	-
Fungal elicitor	BOX-W1	-	1	-	-	-
Wound	WUN	-	-	1	1	-
Anaerobic	ARE	1	1	1	1	-
MejA	TGACG	2	-	-	-	1
	CGTCA	2	-	-	-	1
High transcription level	5UTR Py-rich stretch	1	1	-	-	-
Meristem expression	CAT-BOX	2	-	-	-	-
Endosperm expression	SKn-1	-	2	-	2	-
MYBHv1	CCAAT-box	-	-	1	1	-
Cell cycle regulation	MSA	-	-	1	1	-
Seed specific regulation	RY-element	-	-	2	2	-
Endosperm negative expression	AACA	-	-	-	-	1
Zein metabolism regulation	O2-site	-	1	1	1	2
Anoxic specific inducibility	GC	-	-	-	-	-
Circadian control	Circadian	-	-	1	1	2
Differentiation of the palisade	HD-Zip1	2	-	-	-	-
Leaf morphology development	HD-Zip2	1	-	-	-	-
Protein binding site	HD-Zip3	-	-	-	-	1
	BOXIII	1	1	-	-	-
	ATCT	-	-	-	-	-
	I-box	-	1	2	-	-
	TCT	-	-	-	-	-
	G-BOX	-	-	2	2	-
	GAG	1	-	2	2	-
	GT1	-	-	-	-	1
	ATC	1	-	-	-	-
	1	-	-	-	-	1
	CATT	-	1	-	-	-
	F-box	-	-	-	-	-
Light responsive	Chs-CMA2a	-	-	-	-	-
	GATA	-	1	-	-	-
	ACE	-	-	1	1	-
	BOX4	-	-	1	1	2
	GA	-	-	-	1	-
	LAMP	-	-	2	1	-
	MRE	-	-	1	1	-
	SP1	-	-	1	1	1
	MNF1	-	-	-	-	2
	TGGCA	-	-	-	-	1
	As-2-box	-	-	-	-	-
	AE-BOX	-	-	1	1	-
Promoter & enhancer	CAAT-box	36	28	18	18	18
Core promoter	TATA	10	27	51	51	56

**Table 3:** **Elements present in the promoter region of all isoforms of subfamily II *HKTs *** **in rice according to the PLANT CARE database**

**Function**	**Motif**	***HKT2;1***	***HKT2;1***	***HKT2;2***	***HKT2;3***	***HKT2;4***
		**AB061311**	**AK105132**	**AB061313**	**AJ491820**	**AJ491855**
ABA	ABRE	-	-	-	-	-
	IIB	-	-	-	-	-
	CE3	-	-	-	-	-
Salicylic acid	TCA	1	-	3	-	-
Ethylene	ERE	-	1	-	-	-
Auxin	AuxRR-core	-	-	-	-	-
	TGA	-	-	-	-	-
Gibberellin	P-Box	1	-	-	-	-
	GARE	-	-	-	-	1
MYB	MBS	1	3	-	1	2
Defence&stress	TC-reach	-	2	2	-	-
Heat stress	HSE	-	-	-	-	-
Low temperature	LTR	-	-	-	1	-
Fungal elicitor	BOX-W1	-	-	-	-	-
Wound	WUN	-	-	1	1	-
Anaerobic	ARE	1	-	1	1	-
MejA	TGACG	2	1	-	-	1
	CGTCA	-	1	-	-	1
High transcription level	5UTR Py-rich stretch	-	-	1	-	-
Meristem expression	CAT-BOX	-	-	1	1	-
Endosperm expression	SKn-1	3	2	5	3	4
MYBHv1	CCAAT-box	-	1	-	-	-
Cell cycle regulation	MSA	-	-	-	-	-
Seed specific regulation	RY-element	-	-	-	-	-
Endosperm negative expression	AACA	-	-	-	-	-
Zein metabolism regulation	O2-site	-	-	1	2	2
Anoxic specific inducibility	GC	-	1	-	-	-
circadian control	Circadian	-	1	-	-	-
Differentiation of the palisade	HD-Zip1	1	-	2	-	-
Leaf morphology development	HD-Zip2	1	-	1	-	-
Protein binding site	HD-Zip3	1	-	-	-	-
	BOXIII	-	-	-	1	-
	ATCT	1	-	1	-	-
	I-box	1	1	2	1	1
	TCT	1	-	1	-	-
	G-BOX	-	1	2	-	-
	GAG	-	-	1	2	-
	GT1	-	-	1	2	-
	ATC	-	-	-	-	-
	TGG	-	-	-	-	-
Light responsive	CATT	-	-	-	3	1
	F-box	-	1	-	-	-
	Chs-CMA2a	-	1	-	-	-
	GATA	-	-	-	-	2
	ACE	-	-	-	-	-
	BOX4	-	-	-	-	1
	GA	-	-	-	-	-
	LAMP	-	-	-	-	-
	MRE	-	-	-	-	-
	SP1	2	-	-	-	-
	MNF1	-	-	-	-	-
	TGGCA	-	-	-	-	-
	As-2-box	-	-	-	-	1
	AE-BOX	1	1	1	-	-
Promoter & enhancer	CAAT-box	21	19	39	22	24
Core promoter	TATA	14	1	34	22	54

This analysis showed that the *HKTs* belonging to subfamily I have more of the following motifs than subfamily II: ABA (abscisic acid), auxin, defense responsive, HSE (heat shock element), low temperature response and MYB biding site. However, subfamily II of *HKT* transporters contains the following motifs more frequently than subfamily I: ethylene, gibberellin and salicylic acid response. The results indicated that there are nine ABRE motifs (ABA response element) involved in the response to ABA within subfamily I of HKT promoters, while no ABA response motif is present in subfamily II. ABA is a very important hormone involved in signalling of various stresses such as salinity and drought [29]. The presence of ABRE and MYB motifs were also revealed in *MtATP6* promoter as an abscisic acid-mediated signalling [30]. At salt conditions, It has been also shown that lack of *AtHKT1;1* (*HKT* isoform in *Arabidopsis thaliana*) activation in mutated *Arabidopsis thaliana* leads to Na^+^ accumulation in shoot and its decrease in root, in comparison to control plants [8]. Thus, *AtHKT1;1* has a vital role in Na^+^ exclusion from the shoot to root [31]. *OsHKT1;5* (*HKT1;5* isoform in rice) is an ortholouge of *AtHKT1;1* associated with Na^+^ exclusion from the xylem and has the same role in rice [32]. Nevertheless, In the other study, Kader and co-workers in 2006 showed that the expression of *OsHKT1;1,* another *HKT* isoform in rice, was unexpectedly high in salt sensitive rice cv. BRRI Dhan29 than that in the salt tolerant cv. Pokkali. This result also indicated that in salt sensitive cultivar, *OsHKT1;1* expression happened in earlier sampling time than the salt tolerant one. Therefore, *OsHKT1;1* is vital in Na^+^ influx into plant cell compared to *OsHKT1;5* and *AtHKT1;1*[33]. Altogether, different *HKT* isoforms have variety of expressions and actions at different tissues for unknown reasons [15]. We assume that presence of ABA element in the promoter of these genes can be a reason for salinity tolerance. Interestingly, the core promoter (TATA box), at the position of −50 and −30 nucleotides upstream from start codon, was more frequent in subfamily I than subfamily II. As the function of TATA box is in transcription initiation, increased expression of subfamily I HKT transporters is expected compared to subfamily II. However, according to different studies, it seems that sometimes subfamily II of *HKT* transporters even in salt condition have more expresstion [8]. As a result, it appears that subfamily I primarily may be more critical in many stress responses in plants as its activation is related to Na^+^ transport. On the other hand, it is possible that by continuing stress, the expression of subfamily II will increase, leading to K^+^ transport into the plant cells, and reducing salinity damages [33,34].

*OsHKT2;1*, belonging to subfamily II, exhibited higher expression in salt tolerant cv, Pokkali than salt sensitive cv. BRRI Dhan29, particularly in shoot. Consistent with this result, it is clear that *OsHKT2;1* involves in K^+^ uptake [33]. In this study, Osiris result at *P = 0.05* indicated that all of the significant motifs identified in both subfamilies have ABA response elements. These motifs were observed in *KT1;1*, *HKT1;3,* and *HKT1;4*, which belong to subfamily I *HKTs*, as well as *HKT2;1,* and *HKT2;2,* which are related to subfamily II (Table 4).

**Table 4:** **Significant elements in the promoters of all *HKT *** **isoforms of rice at *P = 0.05 *** **according to the Osiris database**

**Cis-element**	**Locus**	**Gene**	**Function**
ABADESI1, hox1	Os06g0701700	*HKT1;1, HKT2;1, HKT2;2*	ABA response
ABADESI2	Os04g0607600	*HKT1;4*	ABA response
GCrichrepeatIV	Os02g0175000	*HKT1;3*	ABA response

**Figure 1:**
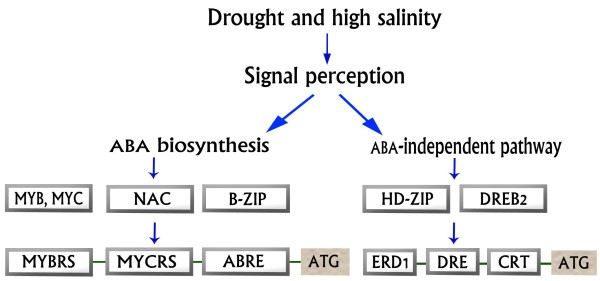
**Different active transcription factors responding to salinity and drought stresses in ABA-dependent and ABA-independent pathways during the induction of stress genes.** Various transcription factors are triggered in response to stresses and mediate their influence on stress genes mainly through two pathways. The transcription factors involved in ABA-dependent pathways are b-ZIP (basic leucin zipper), MYC (myelocytomatosis oncogene) and MYB (myeloblastosis oncogene), NAC (NAM, ATAF1,2 and CUC2), which interact with ABRE (ABA-responsive element) or MYCRS/MYBRS elements in the promoters of stress genes. Members of the ABA-independent pathways include a number of transcription factors, such as HD-ZIP (Homeodomain Leucine Zipper Protein) and DREB2 (DRE-binding protein), which are activated during salt and drought stresses. ATG means start codon, DRE / CRT (dehydration responsive element/C-repeat) and ERD1 (early responsive to dehydration1.

Osiris found the element named Hox1(homeodomain transcription factor) which is a putative member of the leucine-zipper (HD-ZIP) transcription factor class [35] and HD-ZIP which is an ABA-independent motif exist in both subfamilies I and II. Besides the role of ABA in controlling plant responses to salinity, regulating plant responses to drought stress are managed by ABA [36,37]. We suggest a model for motif activation in two pathways related to ABA in Figure 1 in response to drought and salinity stresses since ABA has a central role in both stresses. The next significant element was ABADESI1, which was identified in both *HKT* subfamilies. ABADESI1 is a motif involved in osmotic responses related to physiological processes and is identified with the RAB16-A gene promoter between the −294 and −52 region of this gene; it confers ABA-dependent expression on the chloramphenicol acetyl-transferase reporter gene in rice [38]. There was also a GC-rich repeat IV motif in the *HKT6* promoter. Previous studies have indicated that the GC motif in the RAB 21 gene promoter contains four different GC types at the range of −1 to −200 that are active in response to salinity, drought, and ABA in rice [38]. ABADESI2 is an ABA response element, located between −180 and −160 of the wheat histone H3 promoter that plays an essential role in drought and salinity stresses [39]. Interestingly, *HKT* subfamily II had both ABA-dependent and independent elements on the promoter region.

*HKT1;5,* belonging to subfamily I, significantly up-regulates in salinity condition, especially in *Aegilops crassa*[15]. ABA response elements in subfamily I are more frequent than subfamily II. Consequently, it seems reasonable that this subfamily, even in high salt conditions, is responsible for Na^+^ exclusion rather than K^+^ transport. It appears that the various roles of these subfamilies in K^+^ and Na^+^ transport and their different functions are related to their special architecture of their promoter regions.

### Synteny analysis and identification of novel interacting genes

**Figure 2:**
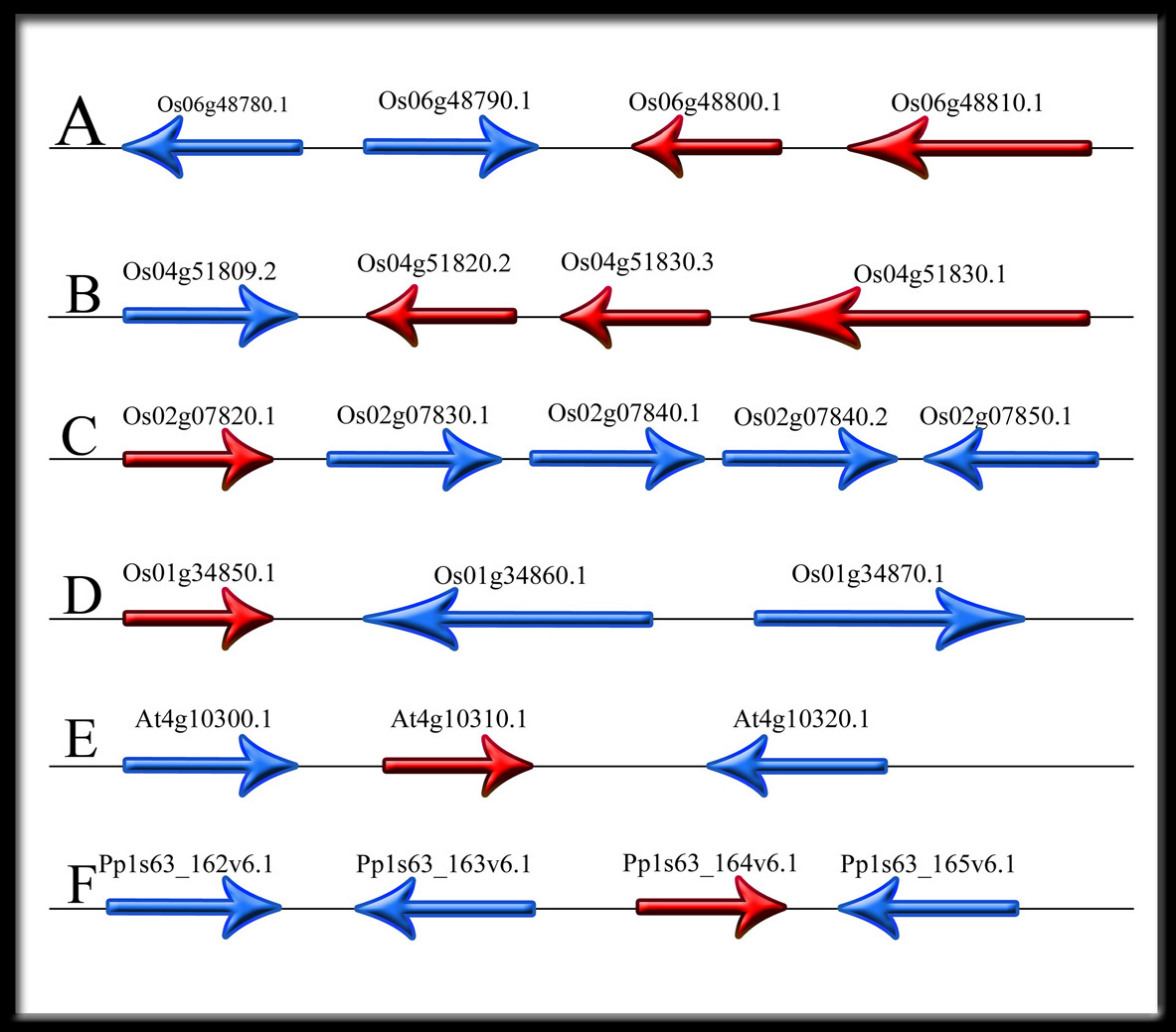
**Gene arrangement revealed by *in silico *synteny analysis of different *HKT *homologues in rice (A to D), *Arabidopsis thaliana *(E) and *Physcomotrell patens *(F) along the chromosome.** The red colour indicates *HKT* homologues along a chromosome, and there are several loci (blue colour), which are largely unidentified, near *HKTs*. The red loci on chromosome 6 of rice are Os06g48800.1 *(HKT2;4)* and Os06g48810.1 *(HKT2;1).* On chromosome 4, Os04g51820.2 and OS04g51830.3 are *HKT1;1*, and OS04g51830.1 is *HKT1;4.* On chromosome 2, Os02g 07820.1 is *HKT1;3*, and on chromosome 1, Os01g34850.1 is *HKT2;3.* In Arabidopsis thaliana, AT4g10310.1 is *HKT1;1*, and in *Physcomotrell patens*, Pp1s63_164v6.1 is *HKT1.*

*In silico* synteny was assessed at rice, *Arabidopsis thaliana* and *Physcomitrella patens HKT* loci (Figure 2). The results indicated that three loci (Os04g51809.2, Os06g48780.1, and Os06g48790.1) exist upstream of the *HKT* isoforms in rice, and there are six loci downstream of the mentioned genes (Os02g07830.1, Os02g07840.1, Os02g07840.1, Os02g07840.2, Os02g07850.1, Os01g34860.1, and Os01g34870.1). It is probable that *HKT* gene neighbours may have a similar function as *HKTs*. The other loci identified based on *in silico* synteny are currently unidentified. As a result, we proposed that these loci may play a role in the response to salt or drought stress, similar to *HKTs* or, likely, other unknown *HKT* isoforms that will be found in the future. It should be noted that genes with similar function usually cluster together along the chromosomes during the evolution. The point is that *HKTs* belonging to subfamily I are not together with subfamily II. Thus, having different and specific cis elements is acceptable in *HKTs* promoter region. Remarkably, analysis of those loci indicated that two loci, Os02g07840.2 and Os01g34860.1, near *OsHKT1;3* and *OsHKT2;3*, respectively, are b-ZIP protein and leucine zipper protein-like. Thus, the result of synteny is in agreement with promoter analysis results showing that ABA response elements are the most important motifs in *HKT* promoters. Therefore, it is possible that ABA can also influence loci close to *HKTs*. Interestingly, *HKT2;1* and *HKT2;4* are located on chromosome 6, and there are three homologs of *HKT1;1* and one of *HKT1;4* neighbouring each other on chromosome 4 (Figure 2). Interestingly, *HKTs* belonging to each subfamily (I or II) are located near to each other on chromosomes.

There is one locus upstream and one locus downstream of the *HKT* gene in *Arabidopsis thaliana*, which are AT4G10300.1 and AT4G10320.1, respectively. *Arabidopsis thaliana* has one *HKT* isoform (*AtHKT1;1*) which is responsible for excluding Na^+^ from phloem to xylem cells [8]. This isoform plays a very important role in salinity tolerance in this plant. *Physcomitrella patent* is a salt-tolerant plant. There are two loci (Pp1s63_162v6.1 and Pp1s63_163v6.1) upstream and one locus (Pp1s63_165v6.1) downstream of the *HKT* gene in this plant (Figure 2). We suggest that these loci probably have an essential duty to improve tolerance under some stresses in *Arabidopsis thaliana* and *Physcomitrella patent*.

The results of Gramene database showed that many genes close to rice *HKTs* are unidentified. The known ones, located 15 kb up- and downstream of *HKTs*, are shown in Table 5 which encode transporters, kinases and proteases. It has been suggested that protein kinases are involved in signal transduction and are activators of *SOS* (salt overlay sensitive) genes [15]. Regarding the remarkable role of kinase proteins in SOS processes under osmotic response [40-42], it is possible that these proteins influence *HKTs* as well.

**Table 5:** ***In silico *** **synteny indicated known upstream and downstream loci of *HKT *** **isoforms in rice using the Gramene database**

**Locus**	**down stream**	**Locus**	**Upstream**	**GENE**
Os01g20160	skc1	Os01g20120	near skc1	*OsHKT1;5*
Os02g07830	Na transporter	Os02g07760	*SSADH*	*OsHKT1;3*
		Os02g07780	AK062651.1	
		Os02g07790	*CTR1*	
Os04g51830	*HKT1;4*	Os04g51820	*HKT1;1*	*OsHKT1;4*
Os04g51950	Kinase			
	(TAP)- kinase	Os06g48520	putative NB-ARC domain-	*OsHKT2;1*
Os06g48650	Protease	Os06g48590	(TAP)- kinase	
		Os06g48770	Serine βlactamse family	*OsHKT2;4*

### Pathway Discovery in *AtHKT1;1* and Identification of Co-expressed Genes

#### Network of *AtHKT1;1* in response to jasmonate and ethylene hormones

**Figure 3:**
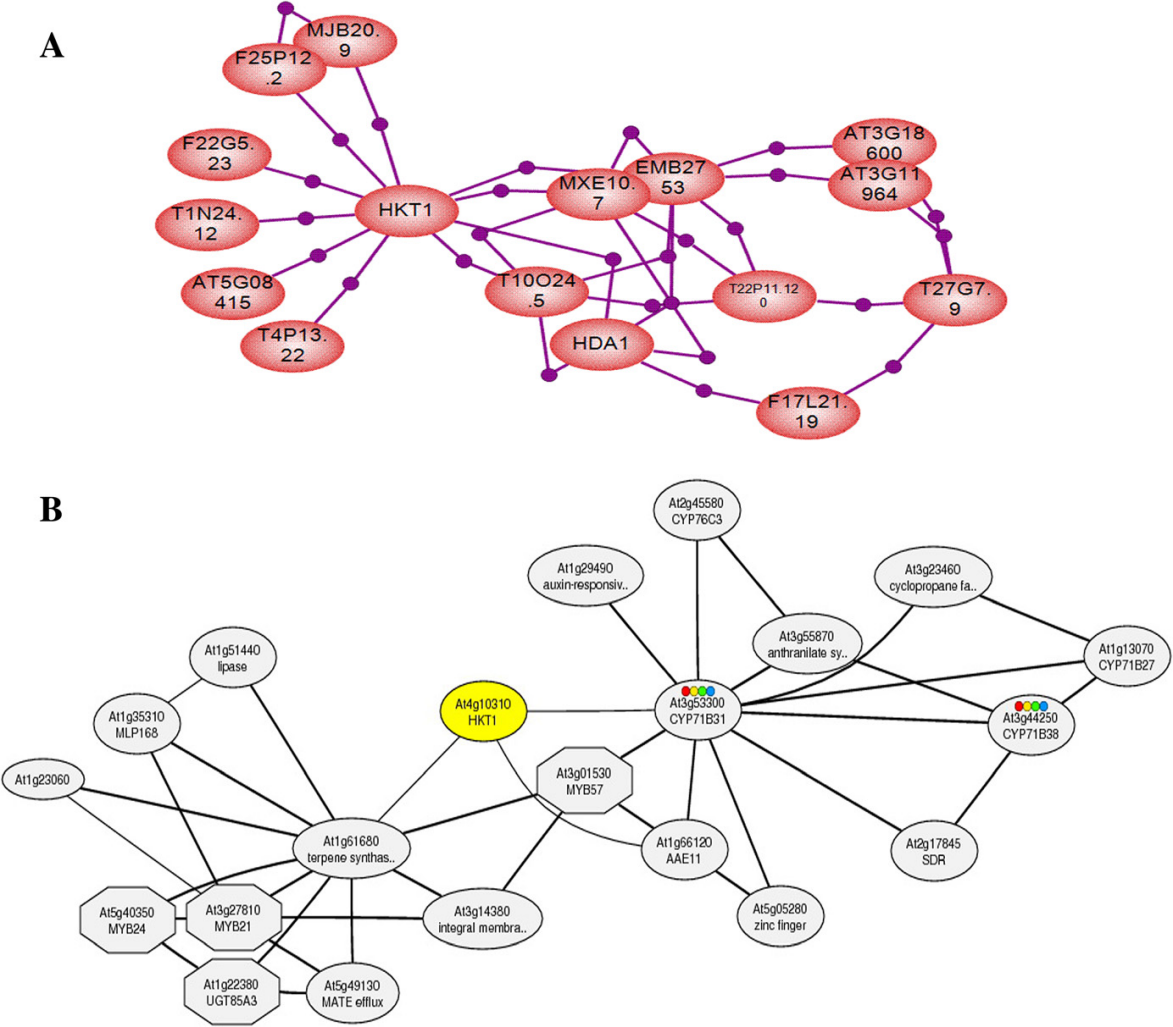
**Pathway of *AtHKT1;1 *and related proteins constructed using pathway studio package. (A)** Down regulated genes in the *AtHKT1;1* protein pathway are shown. These proteins are related to the *AtHKT1;1* protein and are down regulated in the selected microarray experiment, [Microarray ATH1-121501: analyzing crosstalk between jasmonate and ethylene in Col-0, coi1-2, and ein3eil1 strains in two treatments of Mock and MeJA, downloaded from “Plant Expression Database” ( http://www.plexdb.org)]. **(B)** Condition-specific co-expression of genes in the *AtHKT1;1* pathway according to the ATTED-II database ( http://atted.jp). In ATTED co-expression mining, correlation rank is used rather than Pearson correlation value. Source of GeneChip data in ATTED-II database is TAIR http://arabidopsis.org/index.jsp, and 58 experiments and 1388 array slides are used for drawing this co-expressed network. Octagon shapes mean that the genes they cover are transcription factors while ovals cover genes with different functions. Four colourful circles within oval’s of CYP71B31 and CYP71B38 genes are related to various KEGG http://www.genome.jp/kegg/ pathways which these two genes can be active there, so that red shows Naphthalene and anthracene degradation, yellow shows gamma-Hexachlorocyclohexane degradation, green shows Limonene and pinene degradation and blue shows Phenylpropanoid biosynthesis pathways. While the figure A shows down regulated protein associated with *AtHKT1;1*, the up regulated proteins connected to *AtHKT1;1* are shown in figure B.

Based on the selected microarray experiment from “Plant Expression Database” (Microarray ATH1-121501) which evaluates cross-talk between jasmonate and ethylene on Arabidopsis seedlings, network of interacting proteins with *AtHKT1;1* was recognized. The main pathway was so complex making it difficult to identify the nearest proteins connected to *AtHKT1;1*; to detect it easier, the specific pathway associated with proteins in connection with *AtHKT1;1* was predicted separately using pathway studio, as shown in (Figure 3 A).In this pathway, *AtHKT1;1* was down regulated, showing reduction in expression. It appears that using jasmonate and ethylene decreased the function of *AtHKT1;1* in *Arabidopsis* seedlings. The question is, why was *AtHKT1;1* down regulated? As we discussed before, the most important hormone in *HKT* activation process is ABA. This idea was also confirmed through promoter analysis of *HKT* genes. Therefore, it is reasonable that other hormones are not very active rather than ABA where *HKTs* are expressed strongly. On the other hand, combined effect of two hormones can be another reason of decreasing *AtHKT1;1* expression [43]. As a very interesting point, the MeJA response element was found in the promoter region of *TmHKT1;5* as well, indicating that this transcription factor is not convenient in high salinity milieus, and proving the result of network discovery in which the *AtHKT1;1* was down regulated under treatment of MeJA.

However, the results indicated that some of the proteins in the *AtHKT1;1* pathway were involved in ion transporting (Table 6). This result was predictable due to the role of *HKTs* as transporters. Although *AtHKT1;1* was down-regulated in the selected experiment, it seems that other transporter genes were up-regulated instead. Perhaps in this situation (two hormones crosstalk) there was not any need to express *AtHKT1;1*.

**Table 6:** **Results of the pathway discovery analysis indicating proteins activated in the *AtHKT1;1 *** **network based on microarray analysis of cross-talk between jasmonate and ethylene signalling in Arabidopsis seedlings using pathway studio 9**

**Protein**	**Function**
Hda1	Encodes a histone deacetylase involved in jasmonic acid and ethylene-dependent pathogen resistance
MJB20.9	Protein kinase super protein amino acid phosphorylation
ATCHX23	Member of a putative Na+/H + antiporter family
F22G5.23	Arabidopsis thaliana sterol 4-alpha-methyl-oxidase mRNA
EMB2753	Embryo development ending in seed dormancy
T1N24.12	Protein amino acid phosphorylation
T10O24.5	Encodes SIN3-like 6, a homologue of the transcriptional repressor
KCO4	Encodes AtTPK4, a member of the Arabidopsis thaliana K + channel family of AtTPK/KCO proteins
AT5G08415	Radical SAM superfamily protein; FUNCTIONS IN: 4 iron, 4 sulphur cluster binding, lipoic acid
F25P12.2	Hydroxyproline-rich glycoprotein family protein
MXE10.7	N-acetyltransferase activity metabolic process
T4P13.22	Encodes an SNF1-related protein kinase that physically interacts with the SCF subunit SKP1/ASK1 and the 20S proteosome subunit PAD1. It can also interact with PRL1 DWD-containing protein. Based on in vitro degradation assays and cul4cs and prl1 mutants, there is evidence that AKIN10 is degraded in a proteasome-dependent manner and that this depends on a CUL4-PRL1 E3 ligase

### Co-expressed network of *AtHKT1;1*

In addition to prediction the down regulated network of proteins together with *AtHKT1;1*, the genes co-expressed with *AtHKT1;1* were compared to find out what genes are most likely active when *AtHKT1;1* is expressed. In this case, despite the other pathway, the up regulated genes with induced expression are shown. It was found that the three nearest proteins in the above gene pathway were TPS14, CYP71B31 and F15E12.22, which are a terpene synthase, a protein involved in oxidation reduction and an acyl-activating enzyme. However, presence of oxidation reductase protein shows that the possibility of plant toxicity in some stress conditions is obvious, and some proteins like reductase must decline this danger. Additionally, co-expressing oxidation reductase proteins with *AtHKT1;1* attests that the expression of *AtHKT1;1* increases Na^+^ in some cell types, and turns the detoxification system on. Other pathway proteins are shown in (Figure 3 and Table 7).

**Table 7:** **Results of the pathway discovery analysis indicating proteins present and activated in the *AtHKT1;1 *** **pathway in the ATTED database**

**Gene or ID**	**Function**
TPS14	Terpene synthase 14
CYP71B31	Mono oxygenase oxidoredoctasee
F15E12.22	Acyl-activating enzyme 11
F5D21.19	Lipase class 3 family protein
MLP168	Defence response
MYB21 MYB3	Lipase class 3 family protein
MYB24	DNA binding-jasmonic & giberellic acid response
At3g14380	Integral membrane family protein
At5g49130	MATE efflux family protein
AtUGT85A3	Glucuronosyl transferase
CYP76C3	Electron carrier activity
At1g29490	Auxin-responsive family protein
At3g55870	Anthranilate synthase, alpha subunit, putative
AtMYB57	DNA binding-jasmonic & giberellic acid response
At5g05280	Zinc finger (C3HC4-type RING finger) family protein
At2g17845	Short-chain dehydrogenase/reductase (SDR) family protein
At3g23460	Cyclopropane fatty acid synthase-related
At3g44250	Electron carrier/ heme binding / monooxygenase/
At1g13070	Putative cytochrome P450

The designed co-expressed network in this study, based on a large amount of publicly available transcriptomics data, significantly elaborated our understanding of the gene interaction and function of *AtHKT1;1*. Developed databases of gene co-expression analysis which utilize large amount of publicly available transcriptomics data are offering valuable information on relative expression levels of thousands of genes simultaneously which can be exploited for drawing co-expression networks [44]. In fact, large collections of expression data derived from EST, microarray, or RNA-seq platforms contain information about concerted changes in transcript levels these datasets beyond the original purpose of each experiment.

Traditionally, Pearson’s correlation coefficient is index of co-expression where “1” indicates strong Relation and “0” indicates no relation. In this study, we employed Mutual Ranking (MR) which is a more robust co-expression measurement [45]. MR provides a reliable statistical tool for measurement of co-expression between genes in different transcriptomics experiments.

### Regulatory elements on promoter region of *TmHKT1;5* (wild wheat) are more frequent and variable than *TaHKT1;5* (bread wheat)

Statistics of regulatory elements on the promoter region of *TmHKT1;5* and *TaHKT1;5* is presented at Table 8. Interestingly, regulatory elements on the promoter region of *HKT* in wild genotype have more frequency that cultivated genotype (mean of 2.28 regulatory element per unit compared to 1.92 regulatory element per unit). More importantly, regulatory elements are more variable in wild wheat compared to cultivate wheat (variance of 19.54 verses 11.55).

**Table 8:** **Descriptive statistics of regulatory elements on the promoter region of *TmHKT1;5 *** **(wild wheat) compared to *TaHKT1;5 *** **(bread wheat)**

	**Central tendency**		**Variation tendency**			
Gene	**Mean**	**Median**	**StDev**^**1**^	**Variance**	**CV**^**2**^	**Range**
*TmHKT1;5* (wild wheat)	2.28	1.00	4.42	19.54	193.42	22.00
*TaHKT1;5* (bread wheat)	1.92	1.00	3.39	11.55	176.22	18.00

^1^Standard deviation.

^2^Cofficient of variance.

It has been discussed that compared to cultivated genotypes, wild genotypes has better understanding from environment in com environment which helps them to manage energy more efficiently [30]. As example, before stress, wild genotypes keep the expression of mitochondrial ATP synthase lower than cultivated genotypes and save a considerable amount of energy. In contrast, early in stress, a sharp increase in activation and expression of ATP synthase happens in wild genotypes to cope with stress where this expression is much higher than cultivate genotypes [30]. The mentioned highly efficient energy consumption system needs a fast and reliable understanding of environmental conditions and signalling pathways.

Regulatory elements on the promoter region are the central inter-mediators which understand the environmental conditions from one site and activate transcription of genes on the other site. In fact, regulatory elements are the developed model of 2-component systems in bacteria.

It can be concluded that the higher frequency and higher variation of the regulatory elements on the promoter region of wild genotype provides the opportunity to respond to more elicitors and achieve the better understanding of environmental conditions. In other words, high efficiency in arrangement of regulatory elements on the promoter region guaranties higher reaction and better energy management. Additionally, higher frequency of specific regulatory elements such as CAAT-box (Table 1) contributes in rapid activation of transcription by the promoter of wild genotype.

Regarding the facts that up to now, breeding programs have defined solely based on the coding genes and the key roles of non-coding regions such as promoters are neglected [13], it is highly possible that the promoter based resistance mechanisms have not been transferred to cultivated genotypes during breeding programs. Recently, we presented a novel gene discover approach irrespective of gene sequence/blast based on promoter structure. In this approach, organization of regulatory elements was used as a module for mining of the whole genome and discovery of genes with similar promoter architecture [46].

## Conclusion

In this study, analysis of *HKT* transporters was carried by a range of i*n silico* methods. It is likely that specific promoter elements are active in different tissues due to the observed differences in *HKT*s function in various tissues. Identification of regulatory elements helps to illustrate various functions of *HKT* genes in plants. *In silico* synteny and pathway discovery were useful for identification and characterization of *HKT* isoforms and unravelling the molecular networks which they participate.

## Abbreviations

HKT: High affinity potassium transporters.

## Competing interests

The authors declare that they have no competing interests.

## Authors’ contributions

MZB, EE and AN designed, and conducted the analyses. MZB, EE and AN wrote the manuscript. All authors read and approved the final manuscript.
